# Pattern of antimicrobial usage in livestock animals in south-western Nigeria: The need for alternative plans

**DOI:** 10.4102/ojvr.v82i1.816

**Published:** 2015-04-16

**Authors:** Hezekiah K. Adesokan, IfeOluwapo O. Akanbi, Ibikunle M. Akanbi, Ruth A. Obaweda

**Affiliations:** 1 Department of Veterinary Public Health and Preventive Medicine, University of Ibadan, Nigeria; 2 Federal College of Animal Health and Production Technology, Moor Plantation, Ibadan, Nigeria; 3 Department of Veterinary Medicine, University of Ibadan, Nigeria

## Abstract

Resistance to antibiotics has continued to increase, placing future animal and human disease management in real danger. The developing countries characterised by widespread indiscriminate antibiotic use and in which ‘third-generation’ antibiotics are not readily available or affordable are the worst affected. A 3-year (2010–2012) retrospective survey of antibiotic usage in livestock production in three selected states of south-western Nigeria was conducted. Data obtained from eight purposively selected licensed veterinary pharmaceutical sales establishments in the area, based on keeping detailed sales records for the study period, were analysed using Stata Version 12. Results showed that tetracyclines (33.6%), fluoroquinolones (26.5%) and beta-lactams/aminoglycosides (20.4%) constituted the majority of the antibiotics used over the 3 years. The differences in the quantities of antibiotic types used within each antimicrobial class were statistically significant for tetracyclines (*F* = 59.87; *p* < 0.0001) and fluoroquinolones (*F* = 43.97; *p* < 0.0001) but not for beta-lactams/aminoglycosides (*F* = 3.21; *p* = 0.148). Furthermore, antibiotic consumption increased by 40.4% between 2010 and 2012. Although statistically insignificant (*F* = 0.277; *p* = 0.762), the increasing trend across the years was at rates of 23.5% between 2010 and 2011 and 13.8% between 2011 and 2012. In addition, the findings show a significantly higher consumption rate (*t* = 15.21; *df* = 5; *p* < 0.0001) during the rainy (52.5%) than the dry (47.5%) seasons. The current increasing trend in antibiotic usage holds a serious danger for the future and therefore calls for alternative plans to safeguard future livestock production, food security and human health. This becomes more imperative considering emerging resistance against tetracyclines and fluoroquinolones, the foremost remedies for livestock diseases in most developing countries.

## Introduction

The use of antimicrobials for treatment, prophylaxis, metaphylaxis and as growth promoters in food-producing animals is essential for a sustainable and economically viable animal industry (Acar & Röstel 2001; Van Vuuren, Picard & Greyling 2007). However, their use in animal populations has been a particular concern (Aarestrup *et al.* 2001; Klare *et al.* 1999), especially when the drug classes are the same as, or related to, the pharmaceuticals used in the control of human infections. Phillips *et al.* (2004) reported that the use of antibiotics in food animals selects for bacteria resistant to antibiotics used in humans, and these might spread via the food to humans and cause human infection. This is further substantiated by other reports that indicate that certain essential life-saving antimicrobials are becoming less effective, whereas there are fewer alternatives available to treat the diseases for which these antimicrobials are required (Danish Integrated Antimicrobial Resistance Monitoring and Research Programme [DANMAP] 2004; Van den Bogaard & Stobberingh 2000; White *et al.* 2001). Thus, antimicrobial resistance, and the resulting failure of antimicrobial therapies in humans, has become a mounting public health problem of global significance and presents a major and growing threat to effective treatment of bacterial infections (Mather *et al.* 2012).

Besides the adverse effects on humans, resistant variants of microbes, as well as species that are inherently resistant resulting from antimicrobial resistance, can become dominant and spread in host-animal populations. According to previous reports (Acar & Röstel 2001; European Medicines Agency 2011; Marra *et al.* 2010), the use of antibiotics in livestock production is contributing significantly to antibiotic resistance in species of bacteria that are common to humans and animals. The more an antibiotic is used, the more likely resistant populations are to develop amongst pathogens and amongst commensal bacteria of an increasing number of animals in an exposed population. This selection for resistant bacteria in agricultural production environments and the subsequent impact on animal and human health has therefore become a major concern and is the subject of many reports (Institute of Food Technologists 2006).

Whilst policy makers in developed countries debate the merits of limiting antibiotic use in food animals (Food and Drug Administration [FDA] 2010), the potential benefits of such limits could be overshadowed by amplification in developing countries and dissemination of antibiotic-resistant bacteria and resistance traits through travel and trade. Therefore, antibiotic usage and its consequent effects in developing countries such as Nigeria remain a significant focus of attention for industrialised countries as well. However, data on the actual volumes and patterns of antimicrobial usage in animal health, a significant factor for sustainable livestock production, are very scanty in Nigeria. Yet Nigeria's population, expected to reach 402 million people by the year 2050 (Bamaiyi 2013), is growing at a faster rate than the increase in animal products in the country. This study therefore sought to determine the quantities and pattern of antibiotic usage in livestock in south-western Nigeria, with a view to providing insights into the implications of current antimicrobial usage in order to ensure a sustainable livestock industry for the ever-increasing human population in the future.

## Materials and methods

### Study site

This study was carried out in three of the six states in south-western Nigeria. Ibadan, Akure and Osogbo, which are respectively the state capitals of Oyo, Ondo and Osun, were used. These states were selected because of their relatively high livestock activities. They have sizeable expanses of arable land and rich fertile soils that are good for the cultivation of a wide variety of food crops and animal production. Generally, livestock activities in the area are on the increase and there is a high dependence on livestock as a source of employment, revenue and milk and meat production. There are large-scale livestock production industries particularly of cattle, sheep and goats in these areas, mainly in semi-intensive farming systems in which animals are taken out to graze and then returned to their pens later in the evening, besides other small intensive and semi-intensive livestock farming operations that characterise most households. In addition, there is unregulated access to veterinary drugs; a farmer could decide to purchase and administer drugs without veterinary prescription and supervision.

### Data collection and analysis

A total of 10 out of 14 licensed veterinary pharmaceutical sales establishments based on the main inclusion criterion (i.e. the major sales establishments in the area with detailed sales records between 2010 and 2012) were purposively selected; with five, three and two out of seven, four and three from Oyo, Ondo and Osun respectively. These figures respectively represent 71%, 75% and 67% of the veterinary drug sales establishments with detailed sales records in the respective states. Records of the quantities of the different antibiotics sold mainly for use in livestock including cattle, sheep and goats over the 3 years were obtained and expressed as units of frequencies of sales. Monthly sales records of each of the antibiotics were also noted. In addition, the antibiotics were categorised into their different classes. Data analysis was carried out using Stata Version 12. The data collected were coded and the percentages of the various antibiotics sold over the study period were calculated. The rates of increase in the use of antibiotics across the years were calculated by dividing the difference in the quantities consumed between two consecutive years by the quantities consumed in the previous year, which was then multiplied by 100 (to convert it to percentage). Analysis of variance (ANOVA) was used to test for level of significant difference in the quantities of the different classes of antibiotics consumed across the states as well as the study periods. The Student's *t*-test was used to compare the means of the quantities of the different antibiotics sold across the rainy and dry seasons within the study periods.

## Results

Of the ten selected licensed veterinary pharmaceutical sales establishments, only eight (Ibadan = 3; Akure = 3; Osogbo = 2) were willing to participate in the study. In all, a total of 23 234 units of 11 different antibiotics belonging to six antimicrobial classes were sold over the 3 years (Table 1). There was a significant difference in the volumes of different classes of the antimicrobials sold across states (Table 1). Tetracyclines (33.6%) were the most frequently sold antimicrobials, followed by fluoroquinolones (26.5%), beta-lactams/aminoglycosides (20.4%) and macrolides (15.1%). Others were furatadone (2.3%) and chloramphenicol (2.1%) (Table 2). There was a significant difference (*F* = 40.87; *p* < 0.0001) in the quantities of the various classes of antimicrobials sold across the 3 years.

**TABLE 1:** Distribution of antibiotic usage in livestock production in south-western Nigeria with respect to state (2010–2012).

Antibiotic class	Antibiotic type	Quantities sold per year									*F*	*p*-value
		Ibadan			Akure			Osogbo				
		2010	2011	2012	2010	2011	2012	2010	2011	2012		
Tetracyclines	Oxytetracycline	438	478	578	519	510	574	390	470	520	-	-
	Doxycycline	383	368	279	11	11	29	NA	NA	NA	-	-
	Chlortetracycline	227	263	143	146	173	244	220	416	437	-	-
**Class total**	**-**	**1048**	**1109**	**1000**	**676**	**694**	**847**	**610**	**886**	**957**	**5.266**	**0.048**
Beta-lactams/ Aminoglycosides	Penicillin-Streptomycin	259	254	558	135	192	223	300	420	588	-	-
	Gentamycin	349	412	382	14	52	108	140	147	199	-	-
**Class total**	**-**	**608**	**666**	**940**	**149**	**244**	**331**	**440**	**567**	**787**	**8.366**	**0.018**
Fluoroquinolones	Enrofloxacin	687	837	835	62	73	96	557	731	802	-	-
	Ciprofloxacin	99	58	167	NA	NA	NA	NA	NA	NA	52.019	< 0.0001
	Norfloxacin	185	247	201	39	21	29	110	100	220	-	-
**Class total**	**-**	**971**	**1142**	**1203**	**101**	**94**	**125**	**667**	**831**	**1022**	**-**	**-**
Macrolides	Tylosin	313	474	525	217	311	350	310	477	525	2.153	0.197
Chloraphenicol	Florfenicol	72	184	158	18	22	29	NA	NA	NA	ND	-
Furatadone	Furmethonol	185	182	167	NA	NA	NA	NA	NA	NA	ND	-
**Total**	**-**	**3197**	**3757**	**3993**	**1161**	**1365**	**1682**	**2027**	**2761**	**3291**	**23234**	**-**

NA, not available; ND, not done.

**TABLE 2:** Patterns of antibiotic usage in livestock production in south-western Nigeria (2010–2012).

Antibiotic class	Antibiotic type	Quantities sold per year			Total	%	*F*	*p*-value
		2010	2011	2012				
Tetracyclines	Oxytetracycline	1347	1458	1672	4477	19.3	-	-
	Doxycycline	394	379	308	1081	4.6	-	-
	Chlortetracycline	593	852	824	2269	9.8	59.87	0.000
**Class total**	**-**	**2334**	**2689**	**2804**	**7827**	**33.6**	**-**	**-**
Beta-lactams/Aminoglycosides	Penicillin-Streptomycin	694	866	1369	2929	12.6	-	-
	Gentamycin	503	611	689	1803	7.8	3.21	0.148
**Class total**	**-**	**1197**	**1477**	**2058**	**4732**	**20.4**	**-**	**-**
Fluoroquinolones	Enrofloxacin	1306	1641	1733	4680	20.1	-	-
	Ciprofloxacin	99	58	167	324	1.4	43.97	0.000
	Norfloxacin	334	368	450	1152	5.0	-	-
**Class total**	**-**	**1739**	**2067**	**2350**	**6156**	**26.5**	**-**	**-**
Macrolides	Tylosin	840	1262	1400	3502	15.1	-	-
Chloraphenicol	Florfenicol	90	206	187	483	2.1	-	-
Furatadone	Furmethonol	185	182	167	534	2.3	-	-
**Total**	**-**	**6385**	**7883**	**8966**	**23234**	**-**	**0.277**	**0.762**

**FIGURE 1:**
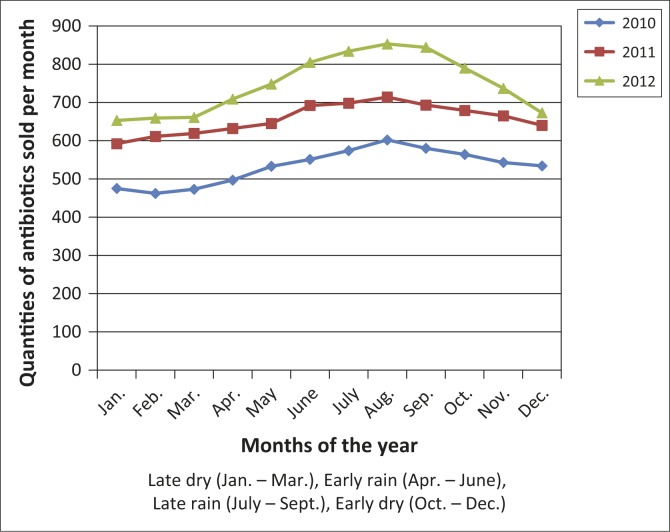
Distribution patterns of antibiotics consumption across the rainy and dry seasons (2010–2012).

Furthermore, the differences in the quantities of antibiotic types within the individual classes of antimicrobials consumed across the years were statistically significant for the tetracyclines (*F* = 59.87; *p* < 0.0001) and fluoroquinolones (*F* = 43.97; *p* < 0.0001), but not for beta-lactams/aminoglycosides (*F* = 3.21; *p* = 0.148). Overall, antibiotic consumption in the study area increased substantially by 40.4% between 2010 and 2012. Although not statistically significant (*F* = 0.277; *p* = 0.762), the increasing trend in the quantities of the antimicrobials sold across the years was at rates of 23.5% between 2010 and 2011 and 13.8% between 2011 and 2012. In addition, it was observed that the level of demand for antibiotics was significantly higher (*t* = 15.21; *df* = 5; *p* < 0.0001) in the rainy (52.5%) than dry (47.5%) seasons (Figure 1).

## Discussion

In the present study, it was documented that tetracyclines (33.6%), fluoroquinolones (26.5%) and beta-lactams/aminoglycosides (20.4%) constituted the majority of antimicrobials used in livestock animal production in south-western Nigeria. The study also found that overall antibiotic consumption increased across the years by 40.4% between 2010 and 2012, whereas the annual livestock growth rate in the area is 2% (Ikhatua 2000). Unlike other antibiotics, the use of tetracyclines, fluoroquinolones, beta-lactams/aminoglycosides and macrolides increased consistently across the years. In addition, a wet-dry season gradient in the quantities of antibiotics consumed over the study period was documented, with significantly higher consumption during the rainy season.

The finding that tetracyclines, fluoroquinolones and beta-lactams/aminoglycosides were the leading antimicrobials used in livestock production in the study area is consistent with a previous report (Adesokan *et al.* 2013), which indicated that these classes of antimicrobials were frequently used in south-western Nigeria. This pattern of antibiotic usage is similar to that reported in Orissa, India by Sahoo *et al.* (2010), where the beta-lactams, fluoroquinolones and tetracyclines were the most commonly used antibiotics in dairy cattle and poultry. Similarly, a survey conducted in South Africa to determine antibiotic usage in food animals showed that tetracyclines and beta-lactams were amongst the first four leading antibiotics commonly used in the country (Eagar, Swan & Van Vuuren 2012). This finding is also corroborated by a report from Tanzania (Katakweba *et al.* 2012), which indicated that the tetracyclines and beta-lactams/aminoglycosides were the foremost antibiotics used in livestock in the area. In Nigeria, these antibiotics are employed by farmers in livestock disease management. For instance, respiratory disease is often managed by using tetracyclines and macrolides, whilst penicillin is used to treat mastitis, metritis, erysipelas in pigs and pneumonia in calves. Generally, tetracyclines are broad-spectrum antibiotics that are active against erlichias, rickettsias, anaplasmas and *Mycoplasma* as well as some protozoa (Prescott 2000). The beta-lactams, particularly penicillin, are employed in the treatment of Gram-positive bacterial infections. In addition, there are a few cases where some antibiotics are used as growth promoters in livestock. However, their uses in general differ from one farmer to the other as they often use their discretion in determining what antibiotics to buy and administer.

Whilst some studies have consistently found low prevalence of resistance to some of these antibiotics, including the fluoroquinolones (Iwalokun *et al.* 2001; Okeke, Fayinka & Lamikanra 2000), an upward trend has recently been observed with these agents. The frequent use of these antibiotics has been linked to the reported emergence of resistant bacterial organisms in both animals and humans in the study area. For instance, Aibinu *et al.* (2007) isolated strains of tetracycline- and beta-lactam-resistant *Escherichia coli* from both animals and humans in south-western Nigeria. Oluyege *et al.* (2009) in a similar study reported various organisms resistant to antimicrobials, including oxytetracycline in cooked food sold on a south-western university campus in Nigeria. In the same vein, Ogunleye, Ajuwape and Adetosoye (2010a, 2010b) reported multidrug resistant *E. coli* and *Salmonella enterica* capable of transferring R factors to these commonly used and often abused antibiotics. Furthermore, Ajayi *et al.* (2011) reported that 64.3% and 67.6% of *E.coli* isolates from cattle in a south-western state were resistant to tetracyclines and aminoglycosides respectively. Also, Olatoye, Amosun and Ogundipe (2012) reported that 91.1% of *E. coli* 0157:H7 isolated from beef in this study area was resistant to tetracyclines. In another report, Daini, Ogbolu and Ogunledun (2005) indicated that resistance to fluoroquinolones in *E. coli* is an increasing problem in Nigeria and other countries.

As previously reported, the development of resistance can significantly shorten the useful lifetime of antimicrobial agents in prophylaxis and therapy of infectious diseases (Sanders *et al.* 1984). Therefore, the emergence of resistance to these antimicrobials, which are the main remedies for livestock disease challenges in most developing countries, particularly Nigeria, presents a serious concern for the future livestock industry. This becomes worse as the majority of livestock animals are domiciled in rural settings amongst farmers who might not be able to afford buying third-generation antibiotics, even if these are available. According to a recent report (World Health Organization [WHO] 2014), there is a need for urgent coordinated action by many stakeholders; otherwise, the world is headed for a post-antibiotic era where common infections and minor injuries that were treatable in the past could once again kill. The need for alternative management measures for livestock diseases in the future therefore becomes urgent.

Furthermore, the study observed an overall relative increasing rate of 40.4% in antibiotic consumption between 2010 and 2012, with specific rates of 23.5% between 2010 and 2011 and 13.8% between 2011 and 2012. This relative increase far exceeds the annual livestock growth rate of 2% previously reported in the area (Ikhatua 2000). This finding suggests that the increasing antibiotic consumption rate in the area was driven by factors other than simply increases in animal population. Similarly, a study by Van Boeckel *et al.* (2014) to determine the global antibiotic consumption in humans showed that antibiotic consumption increased substantially in developing countries – a similar trend as seen in livestock in this study area. This is a matter of concern for livestock disease management and livestock production in general given the existing emergence of bacterial strains resistant to major antibiotics. This pattern of antibiotic consumption follows lack of regulatory control in the sales of veterinary drugs in most developing countries. Whilst Nigerian veterinary legislation forbids any person who is not a registered veterinarian to administer these drugs (National Agricultural Extension and Research Liaison Services 2000), its enforcement is close to zero; hence farmers are often seen purchasing and administering these drugs by themselves. Antibiotics are widely available over the counter and mostly used without prescription (Ezenduka *et al.* 2011). Withdrawal periods are not usually observed and residues have been reported in tissues of food animals and their products (Adesokan *et al.* 2013; Kabir *et al.* 2004). In addition, it is common practice amongst most farmers to administer these antibiotics repeatedly against infections that appear non-responsive to the normal dose they must have given earlier (Nigerian farmers, pers. comm., 15 October 2012), whereas such non-responsiveness might indicate cases of resistant bacterial strains. Again, they often combine various antibiotic types with the same active principles but from different manufacturers, on grounds of perceived effectiveness. Such a practice is substantiated by the report that most antibiotics are used unnecessarily in commercially driven agriculture and when uncertain of a diagnosis or treating largely self-limiting bacterial or viral infections (Laxminarayan *et al.* 2013).

The observed difference in the relative quantities of antibiotics consumed in the rainy and dry seasons is likely to be associated with higher incidence of infections that often characterises the rainy season. A similar seasonal pattern of antibiotic use was noted in a study in India (Sahoo *et al.* 2010), indicating that antibiotic prescription followed the seasonal pattern of occurrence of diseases. In addition, studies from Europe and the United States have reported on geographical variation in antibiotic consumption and resistance, considering the influence of social and climatological factors (Blanch *et al.* 2003; Garcia-Rey *et al.* 2004; McCormick *et al.* 2003; Perez-Trallero *et al.* 2005). Furthermore, a study carried out in Nigeria (Ohaeri 2010) showed that trypanosomosis, which is one of the major livestock diseases in the study area, was significantly higher during the rainy than during the dry season. In addition, a report indicated that the dry season in the study area was characterised by lower vector abundance and infection rates (Ohaeri 2005). Similarly, Fabiyi and Adeleye (1982) reported that the pattern of distribution of fasciolosis, another common disease in livestock in the area, coincided with areas of high rainfall. Generally, the heat intensity during the dry season makes the environment less conducive to most disease vectors’ survival or life-cycle completion (Lawal-Adebowale 2012). This assertion is supported by the report that increased temperature and moisture will enhance disease transmission (Food and Agriculture Organization [FAO] 2002). A similar report (Gale *et al.* 2009) indicated that warmer and wetter weather (particularly warmer winters/rainy seasons as a result of climate change) will increase the risk and occurrence of animal diseases, as certain species that serve as disease vectors, such as biting flies and ticks, are more likely to survive year-round. Measures to reduce or control vector activities should therefore be employed instead of increasingly indiscriminate use of antibiotics during the rainy season.

The findings above notwithstanding, this study had some limitations. Firstly, only three out of six states in south-western Nigeria were used. A higher number could probably have given better insights into the pattern of antibiotics usage in the study area. The authors believe, however, that the antibiotic consumption trends observed in this study could be representative of the situation for all other veterinary pharmaceutical establishments in the south-western states, with similar implications for future livestock production and industry. Secondly, this study was limited to only antibiotics used in mammalian livestock and did not capture those used in poultry, or other veterinary drugs such as antihelminthics, which also have implications for livestock production. Future research is recommended on these areas.

Despite the limitations, this study provides essential information that could safeguard future livestock production for all stakeholders in both the livestock industry and pharmaceutical companies. The patterns of antibiotic usage observed in this study require the relevant authorities to work towards limiting indiscriminate use of antibiotics in animals. In addition, the current situation and trends call for all stakeholders to devise alternative plans in order to safeguard future livestock production. It is also recommended that farmers should be educated on alternative ways of controlling disease, such as limiting vector populations, particularly during rainy season, rather than relying on increased antibiotic use to combat diseases in animals. This is achievable through regular training for farmers organised by the Federal Livestock Department of Nigeria and similar bodies as well as providing necessary materials and tools required for limiting vector populations. The findings from this study might apply to other sub-Saharan African countries, given the unregulated purchase and indiscriminate antibiotic use that characterise these regions. Therefore there is a need for both national and international stakeholder interventions to develop alternative plans to safeguard future livestock production and human health and ensure food security.
